# Involvement of Genetic Factors in Multiple Sclerosis

**DOI:** 10.3389/fncel.2020.612953

**Published:** 2020-12-01

**Authors:** Laura Ferrè, Massimo Filippi, Federica Esposito

**Affiliations:** ^1^Neurology Unit, Istituto di Ricovero e Cura a Carattere Scientifico San Raffaele Scientific Institute, Milan, Italy; ^2^Neurorehabilitation Unit, Istituto di Ricovero e Cura a Carattere Scientifico San Raffaele Scientific Institute, Milan, Italy; ^3^Laboratory of Human Genetics of Neurological Disorders, Institute of Experimental Neurology, Division of Neuroscience, Istituto di Ricovero e Cura a Carattere Scientifico San Raffaele Scientific Institute, Milan, Italy; ^4^Vita-Salute San Raffaele University, Milan, Italy; ^5^Neurophysiology Unit, Istituto di Ricovero e Cura a Carattere Scientifico San Raffaele Scientific Institute, Milan, Italy; ^6^Neuroimaging Research Unit, Institute of Experimental Neurology, Division of Neuroscience, Istituto di Ricovero e Cura a Carattere Scientifico San Raffaele Scientific Institute, Milan, Italy

**Keywords:** multiple sclerosis, genetic factors, inflammation, neurodegeneration, disease severity, treatment response

## Introduction

Multiple sclerosis (MS) is the most common chronic inflammatory disease of the central nervous system (CNS) that affects young adults between 20 and 40 years of age, with a higher prevalence in females (female-to-male ratio 3:1) (Dilokthornsakul et al., 2016). Even though its etiology is still debated, it has historically been considered an immune-mediated disease, whose histopathological hallmarks are inflammation, demyelination, and neurodegeneration (Frohman et al., 2006; Lassmann, 2018). Indeed, acute demyelinating lesions characterized by blood brain barrier breakdown, lymphocyte infiltration, oligodendrocyte loss and astrocytic activation, mainly located in myelin-rich white-matter areas, are distinctive of the disease. Nonetheless, in more recent years it became clear that axonal loss and primary demyelination in the absence of acute inflammatory infiltrates are also present since the earliest stages of the disease (Henderson et al., 2009), challenging the idea that neurodegeneration is secondary to CNS inflammation (Louapre and Lubetzki, 2015). As a matter of fact, it is still unclear whether neurodegeneration is a direct consequence of inflammatory CNS injury or whether it represents a primitive independent process, and a better understanding of the interplay between these two aspects of the disease is warranted. In this perspective, studies investigating genetic factors associated with MS onset and progression can help to shed light on this matter; in fact, compared to cross-sectional histopathological studies that do not permit to establish temporal and/or causal relationship, genetic association studies allow to infer causality and to pinpoint molecular pathways and cell types that play a key role in MS pathogenesis.

## HLA Association

The first MS-associated genetic risk locus discovered in 1972 was located in the human leukocyte antigen (HLA) class I region on chromosome 6 (Jersild et al., 1972; Naito et al., 1972). Since then, several HLA class I and II alleles have been associated with an increased risk of developing MS (Hauser et al., 1989; Patsopoulos et al., 2013) or were found to be protective (Fogdell-Hahn et al., 2000).

The strongest association has been demonstrated with the HLA DRB1^*^1501 allele (Hauser et al., 1989; Oksenberg et al., 2004; Patsopoulos et al., 2013) that confers an almost 3-fold increased risk of MS. The most recent genome-wide association study (GWAS) in MS (Patsopoulos et al., 2019) identified up to 32 independent MS risk-variants in the major histocompatibility complex (MHC) region and, overall, the MHC locus alone is estimated to explain 20% of MS heritability (Patsopoulos et al., 2019).

The HLA locus maps on chromosome 6p21.3 and encompasses more than 200 genes, with important roles in maturation and regulation of the T cell compartment as well as in other immunological processes, supporting the hypothesis that MS is an immune-mediated disease arising from a dysregulation of the peripheral immune system which targets the CNS. Consistently, a fine-mapping study of the MHC region (Patsopoulos et al., 2013) has shown that some of the MS-associated variants in this locus affect the amino-acidic sequence of the peptide-binding groove, potentially influencing antigen recognition and T-cell repertoire specificity.

## Genome-Wide Association Studies of MS Susceptibility

Since 1970s, the knowledge of MS genetic architecture has extraordinarily advanced and several GWAS have been performed, identifying hundreds of additional MS genetic risk variants outside the MHC locus (The International Multiple Sclerosis Genetics Consortium et al., 2007; Bahlo et al., 2009; Baranzini et al., 2009; Sanna et al., 2010; Patsopoulos et al., 2011, 2019; Sawcer et al., 2011; Beecham et al., 2013). GWAS are particularly helpful in the discovery of novel disease variants, because they do not depend on “a priori” assumption on disease pathogenesis and can help to unravel novel molecular mechanisms underlying the disease.

The first MS GWAS (The International Multiple Sclerosis Genetics Consortium et al., 2007) was performed on 931 family trios and identified variants in the interleukin-7 receptor (IL7R) and in the interleukin-2 receptor (IL2RA) loci (Gregory et al., 2007; Weber et al., 2008), again pointing to the crucial role played by the immune system. In the following years, the collaborative efforts of the International MS Genetic Consortium (IMSGC) allowed to combine MS cohorts from several countries, improving the statistical power and leading to a remarkable increase in the number of the identified MS risk variants. In 2011, the analysis of 9,772 MS cases and 17,376 controls from 15 different countries, followed by a replication step in 4,218 cases and 7,296 healthy controls (Sawcer et al., 2011), allowed to confirm most of the previously reported associations and to detect 29 novel risk variants. Noteworthy, the prioritized effects were enriched in genes involved in lymphocyte functions and in particular in T cell activation and proliferation. Moreover, genes linked to Vitamin D metabolism, such as CYP27B1, CYP24A1, and genes coding for targets of MS immune-modulatory therapies, including VCAM1 for natalizumab and IL2RA for daclizumab, were also highlighted. More recently, the greatest advancement in MS genetics field was made possible by a meta-analysis involving 47,351 MS subjects and 68,284 healthy controls (Patsopoulos et al., 2019) that increased the number of MS-associated variants up to 200 autosomal SNPs outside the MHC region and one variant on the X chromosome. Once again, the identified variants seem to be enriched in loci which are active in immune cells, both from the adaptive (T and B cells) and the innate (natural killer and dendritic cells) immune compartments; conversely there was no enrichment of MS susceptibility loci in CNS tissues and, also when analyzing induced pluripotent stem cell derived neurons and purified human astrocytes or microglia, the only significant enrichment was detected in microglial cells, suggesting that also CNS resident immune compartment could play a role in the disease. Similarly, when assessing the effect of the selected variants on gene expression modulation in specific tissues (eQTL effect) and when evaluating the biological pathways enriched in MS genes, findings were indicative of the central role of immune cells, while putative functional consequences in neurons and other CNS cells need further investigations. A more recent study (Factor et al., 2020) applied a so-called “outside variant approach” to identify additional risk variants that physically interact with MS-associated genes and to pinpoint cell-types in which these genes exert their pathogenic role. As expected, the majority of MS loci were predicted to act in T cells, but also B cells and myeloid cells were involved, underlining the role of both the innate and adaptive immune system. Moreover, the authors also identified 6 loci that were predicted to act in the CNS and they hypothesized that a dysregulation of transcriptional elongation in oligodendrocytes could contribute to MS pathogenesis.

Finally, the main role played by the immune system is also reflected in the significant proportion of MS risk variants that is shared with other autoimmune diseases (Richard-Miceli and Criswell, 2012), such as type 1 diabetes, rheumatoid arthritis and intestinal bowel diseases, even though sometimes with opposite effect. The common background with other immunological conditions seems to imply the existence of a genetic basis predisposing to peripheral immune dysregulation; other risk factors, such as environmental exposures and/or infections, could then play a role in determining the target tissue and phenotypic expression of this autoimmune predisposition.

## The Role of Low-Frequency/Rare Variants

Classic GWAS take advantage of very large numbers of enrolled subjects to screen hundreds of thousands common variants over the entire genome but are largely underpowered to detect association with low-frequency and rare variants. More recently, some studies investigated the contribution of rare variants in MS heritability using a candidate gene approach or a whole exome analysis in sporadic and familiar MS cases. Specifically, a study conducted in about 68,000 subjects identified four novel variants driving MS risk independently of common-variant signals, which are implicated in regulatory T cell homeostasis and regulation, IFN gamma biology, and NFkB signaling (Mitrovič et al., 2018). Another study identified an increased burden of rare variants with high predicted pathogenicity in MS cases compared to controls when analyzing genes of the inflammasome pathway (e.g., NLRP1/3, CASP1) (Vidmar et al., 2019). Similarly, an additional work on 34 multi-incident MS families also identified some putative risk variants implicating the inflammasome pathway (NLRP12), as well as other biological processes involved in innate immunological responses, again stressing the role of immune factors in the disease (Vilariño-Güell et al., 2019).

## Genetic Factors Influencing Disease Severity

Overall, genetic studies on MS susceptibility seem to denote a major role for immune-related processes in disease pathogenesis; however, these studies do not take into consideration the highly variable degree of neurodegeneration and disability accumulation that characterize the disease. For this reason, genetic studies investigating variants associated with disease severity represent a complementary approach in understanding MS pathological basis.

First of all, candidate gene studies have assessed the role of genes implicated in MS susceptibility in determining disease course, failing to identify any significant association (Jensen et al., 2010) except for the HLA-DRB1^*^1501 allele (Hauser et al., 2000; Barcellos et al., 2003). Interestingly enough, a subsequent GWAS (Baranzini et al., 2009) also failed to identify an enrichment of associated genes implicated in immunological functions. On the contrary, when disease severity was assessed using MRI measures such as brain volume and T2 lesion load, there was an over-representation of genes related to neural processes (e.g., GRIN2A, NLGN1) such as glutamate signaling and axon guidance. Similarly, a network analysis on results from a GWAS that used brain glutamate concentrations as endophenotype (Baranzini et al., 2010) identified a module enriched in genes implicated in glutamate biology that correlated with the level of brain atrophy and N-Acetylaspartate decline over time, pointing to a possible role of excitotoxicity in modulating disease severity.

Furthermore, an additional GWAS (Briggs et al., 2011) that investigated variants associated with disease severity using the MS severity score (MSSS) and in which a pathway analysis was applied showed an enrichment of genes involved in axon guidance and signaling and neuronal processes as well as with interferon α/β receptor binding and antigen processing and presentation.

## Pharmacogenetic Studies

Finally, also works investigating genetic determinants of response to disease modifying treatments can add to the knowledge of the biological basis of the disease.

Interferonβ (IFNβ) was the first drug to be approved for MS and is also the most investigated in pharmacogenomic studies; specifically, the first of these studies focused on genes known to be implicated in its mechanism of action applying a candidate gene approach. At least 15 genes associated with IFNβ response were identified (Cunningham et al., 2005; Martínez et al., 2006; Gross et al., 2011; Kulakova et al., 2012), although not all the associations have been validated in independent cohorts. Not surprisingly, associated polymorphisms mapped to region encoding type I IFN receptor (Cunningham et al., 2005), IFN response-element sequences, IFN regulatory transcription factors (Gross et al., 2011) and to loci encoding different cytokine genes (IL10, IFNγ) (Martínez et al., 2006; Kulakova et al., 2012). Candidate studies also evaluated the role of the HLA class II locus, and mainly of the HLA DR2 haplotype, in influencing IFNβ response, but no significant association was found (Gross et al., 2011).

More interesting were the results of GWAS on response to IFNβ (Byun et al., 2008; Comabella et al., 2009; Esposito et al., 2015; Clarelli et al., 2017; Mahurkar et al., 2017); these investigations not only confirmed the role of genes implicated in the IFNβ pathway, such as IFNAR2, but also identified genes involved in neuronal functions like GPC5 (Byun et al., 2008), the glutamate receptors GRIA3 (Comabella et al., 2009), GRM3 and GRIK2 (Clarelli et al., 2017) and SLC9A9 (Esposito et al., 2015), which has been linked to neuronal excitability (Gu et al., 2001), pointing to a possible role for glutamate metabolism and excitotoxicity in modulating drug response. Similarly, candidate gene studies on response to glatiramer acetate (GA) (Fusco et al., 2001; Grossman et al., 2007; Tsareva et al., 2012; Kulakova et al., 2017) focused mainly on immune-related genes and found that the clinical outcome during GA treatment is associated with HLA class II genes (Fusco et al., 2001; Gross et al., 2011) and other genes involved in T cell activation (TCRB, Grossman et al., 2007), antigen presentation (CD86) or proinflammatory signaling [(IL1R1 and IL12RB2, Grossman et al., 2007), (CCR5, Tsareva et al., 2012)]. Additionally, a recent GWAS on more than 2,500 GA-treated MS patients (Ross et al., 2017) selected a set of 4 SNPs mapping to HLA-DQB2, MBP, UVRAG, and ZAK, which distinguished signature-positive or negative patients, signature-positive subjects displaying a better outcome over signature-negative individuals.

## Discussion

The results of genetic studies on MS susceptibility (The International Multiple Sclerosis Genetics Consortium et al., 2007; Patsopoulos et al., 2019) identified a strong enrichment of genes and biological processes implicated in immune functions, suggesting that a dysregulation of immune responses, promoted by the genetic background and potentially triggered by environmental factors, is the main mechanism of the disease. Overall, apart from a single study that predicted 6 MS risk loci to be mainly active in CNS cells, where they can disrupt oligodendrocyte maturation (Factor et al., 2020), no particular enrichment in genes and pathways involved in CNS development and regulation was found, implying that neurodegeneration is more likely to derive from an inflammatory insult than to be a primary process itself. However, these studies mainly enrolled patients with a relapsing-remitting form of the disease, which is characterized by a high level of inflammatory activity. Thus, it is possible that genetic variants affecting the degree of neuronal damage and disability accumulation were not adequately picked-up by such studies. Indeed, genetic studies that focused on disease severity showed genes involved in neuronal processes, and in particular in glutamate biology, as associated with the degree of disability progression and neurodegeneration (Baranzini et al., 2009, 2010). Noteworthy, some of these genes and pathways were also associated with response to MS treatments (Comabella et al., 2009; Esposito et al., 2015; Clarelli et al., 2017). Unfortunately, most of the genetic studies on MS severity and treatment response have been performed in relatively modest datasets and lack a validation in larger, independent cohorts due to the difficulty in obtaining detailed longitudinal clinical information in huge cohorts. Nonetheless, these results seem to suggest that inflammation is not the only determinant of disease course and also neuronal factors can modulate the level of neurodegeneration and disability progression in MS. Further studies on larger datasets are required to validate these results and to help identifying potential therapeutic targets to halt neurodegeneration in MS.

## Author Contributions

LF: design and conceptualization of the study and manuscript writing. MF: revision of the manuscript for intellectual content. FE: conceptualization of the study and revision of the manuscript for intellectual content. All authors contributed to the article and approved the submitted version.

## Conflict of Interest

MF is Editor-in-Chief of the Journal of Neurology; received compensation for consulting services and/or speaking activities from Bayer, Biogen Idec, Merck-Serono, Novartis, Roche, Sanofi Genzyme, Takeda, and Teva Pharmaceutical Industries; and receives research support from Biogen Idec, Merck-Serono, Novartis, Roche, Teva Pharmaceutical Industries, Italian Ministry of Health, Fondazione Italiana Sclerosi Multipla, and ARiSLA (Fondazione Italiana di Ricerca per la SLA). FE has received compensation for consulting services and/or speaking activities from Novartis, Sanofi Genzyme, Almirall, TEVA and Merck-Serono. The remaining author declares that the research was conducted in the absence of any commercial or financial relationships that could be construed as a potential conflict of interest.

## References

[B1] Bahlo M. Booth D. R. Simon A. Broadley B. Foote S. J. Griffiths L. R.. (2009). Genome-wide association study identifies new multiple sclerosis susceptibility loci on chromosomes 12 and 20. Nat. Genet. 41, 824–828. 10.1038/ng.396 19525955

[B2] Baranzini S. E. Srinivasan R. Khankhanian P. Okuda D. T. Nelson S. J. Matthews P. M.. (2010). Genetic variation influences glutamate concentrations in brains of patients with multiple sclerosis. Brain 133, 2603–2611. 10.1093/brain/awq192 20802204 PMC2929334

[B3] Baranzini S. E. Wang J. Gibson R. A. Galwey N. Naegelin Y. Barkhof F.. (2009). Genome-wide association analysis of susceptibility and clinical phenotype in multiple sclerosis. Hum. Mol. Genet. 18, 767–778. 10.1093/hmg/ddn388 19010793 PMC4334814

[B4] Barcellos L. F. Oksenberg J. R. Begovich A. B. Martin E. R. Schmidt S. Vittinghoff E.. (2003). HLA-DR2 dose effect on susceptibility to multiple sclerosis and influence on disease course. Am. J. Hum. Genet. 72, 710–716. 10.1086/367781 12557126 PMC1180245

[B5] Beecham A. H. Patsopoulos N. A. Xifara D. K. Davis M. F. Kemppinen A. Cotsapas C.. (2013). Analysis of immune-related loci identifies 48 new susceptibility variants for multiple sclerosis. Nat. Genet. 45, 1353–1360. 10.1038/ng.2770 24076602 PMC3832895

[B6] Briggs F. B. S. Shao X. Goldstein B. A. Oksenberg J. R. Barcellos L. F. De Jager P. L. (2011). Genome-wide association study of severity in multiple sclerosis. Genes Immun. 12, 615–625. 10.1038/gene.2011.34 21654844 PMC3640650

[B7] Byun E. Caillier S. J. Montalban X. Villoslada P. Fernández O. Brassat D. (2008). Genome-wide pharmacogenomic analysis of the response to interferon beta therapy in multiple sclerosis. Arch. Neurol. 65, 337–344. 10.1001/archneurol.2008.47 18195134

[B8] Clarelli F. Liberatore G. Sorosina M. Osiceanu A. M. Esposito F. Mascia E.. (2017). Pharmacogenetic study of long-term response to interferon-β treatment in multiple sclerosis. Pharmacogenomics J. 17, 84–91. 10.1038/tpj.2015.85 26644207

[B9] Comabella M. Craig D. W. Morcillo-Suárez C. Río J. Navarro A. Fernández M.. (2009). Genome-wide scan of 500 000 single-nucleotide polymorphisms among responders and nonresponders to interferon beta therapy in multiple sclerosis. Arch. Neurol. 66, 972–978. 10.1001/archneurol.2009.150 19667218

[B10] Cunningham S. Graham C. Hutchinson M. Droogan A. O'Rourke K. Patterson C.. (2005). Pharmacogenomics of responsiveness to interferon IFN-beta treatment in multiple sclerosis: a genetic screen of 100 type I interferon-inducible genes. Clin. Pharmacol. Ther. 78, 635–646. 10.1016/j.clpt.2005.08.018 16338279

[B11] Dilokthornsakul P. Valuck R. J. Nair K. V. Corboy J. R. Allen R. R. Campbell J. D. (2016). Multiple sclerosis prevalence in the United States commercially insured population. Neurology 86, 1014–1021. 10.1212/WNL.0000000000002469 26888980 PMC4799713

[B12] Esposito F. Sorosina M. Ottoboni L. Lim E. T. Replogle J. M. Raj T.. (2015). A pharmacogenetic study implicates SLC9a9 in multiple sclerosis disease activity. Ann. Neurol. 78, 115–127. 10.1002/ana.24429 25914168

[B13] Factor D. C. Barbeau A. M. Allan K. C. Hu L. R. Madhavan M. Hoang A. T.. (2020). Cell type-specific intralocus interactions reveal oligodendrocyte mechanisms in MS. Cell 181, 382–395.e21. 10.1016/j.cell.2020.03.002 32246942 PMC7426147

[B14] Fogdell-Hahn A. Ligers A. Grønning M. Hillert J. Olerup O. (2000). Multiple sclerosis: a modifying influence of HLA class I genes in an HLA class II associated autoimmune disease. Tissue Antigens 55, 140–148. 10.1034/j.1399-0039.2000.550205.x 10746785

[B15] Frohman E. M. Racke M. K. Raine C. S. (2006). Multiple sclerosis–the plaque and its pathogenesis. N. Engl. J. Med. 354, 942–955. 10.1056/NEJMra052130 16510748

[B16] Fusco C. Andreone V. Coppola G. Luongo V. Guerini F. Pace E.. (2001). HLA-DRB1^*^1501 and response to copolymer-1 therapy in relapsing-remitting multiple sclerosis. Neurology 57, 1976–1979. 10.1212/WNL.57.11.1976 11739812

[B17] Gregory S. G. Schmidt S. Seth P. Oksenberg J. R. Hart J. Prokop A.. (2007). Interleukin 7 receptor α chain (IL7R) shows allelic and functional association with multiple sclerosis. Nat. Genet. 39, 1083–1091. 10.1038/ng2103 17660817

[B18] Gross R. Healy B. C. Cepok S. Chitnis T. Khoury S. J. Hemmer B.. (2011). Population structure and HLA DRB1 1501 in the response of subjects with multiple sclerosis to first-line treatments. J. Neuroimmunol. 233, 168–174. 10.1016/j.jneuroim.2010.10.038 21115201

[B19] Grossman I. Avidan N. Singer C. Goldstaub D. Hayardeny L. Eyal E.. (2007). Pharmacogenetics of glatiramer acetate therapy for multiple sclerosis reveals drug-response markers. Pharmacogenet. Genomics 17, 657–666. 10.1097/FPC.0b013e3281299169 17622942

[B20] Gu X. Q. Yao H. Haddad G. G. (2001). Increased neuronal excitability and seizures in the Na + /H + exchanger null mutant mouse. Am. J. Physiol. Cell Physiol. 281, C496–C503. 10.1152/ajpcell.2001.281.2.C496 11443048

[B21] Hauser S. L. Fleischnick E. Weiner H. L. Marcus D. Awdeh Z. Yunis E. J.. (1989). Extended major histocompatibility complex haplotypes in patients with multiple sclerosis. Neurology 39, 275–277. 10.1212/WNL.39.2.275 2783768

[B22] Hauser S. L. Oksenberg J. R. Lincoln R. Garovoy J. Beck R. W. Cole S. R. (2000). Interaction between HLA-DR2 and abnormal brain MRI in optic neuritis and early MS. Neurology 54, 1859–1861. 10.1212/WNL.54.9.1859 10802800

[B23] Henderson A. P. D. Barnett M. H. Parratt J. D. E. Prineas J. W. (2009). Multiple sclerosis: distribution of inflammatory cells in newly forming lesions. Ann. Neurol. 66, 739–753. 10.1002/ana.21800 20035511

[B24] Jensen C. J. Stankovich J. Van der Walt A. Bahlo M. Taylor B. V. van der Mei I. A. F.. (2010). Multiple sclerosis susceptibility-associated SNPs do not influence disease severity measures in a cohort of Australian MS patients. PLoS ONE 5:e10003. 10.1371/journal.pone.0010003 20368992 PMC2848851

[B25] Jersild C. Svejgaard A. Fog T. (1972). HL-A antigens and multiple sclerosis. Lancet 1, 1240–1241. 10.1016/S0140-6736(72)90962-2 4113225

[B26] Kulakova O. Bashinskaya V. Kiselev I. Baulina N. Tsareva E. Nikolaev R.. (2017). Pharmacogenetics of glatiramer acetate therapy for multiple sclerosis: the impact of genome-wide association studies identified disease risk loci. Pharmacogenomics 18, 1563–1574. 10.2217/pgs-2017-0058 29095108

[B27] Kulakova O. G. Tsareva E. Y. Boyko A. N. Shchur S. G. Gusev E. I. Lvovs D.. (2012). Allelic combinations of immune-response genes as possible composite markers of IFN-β efficacy in multiple sclerosis patients. Pharmacogenomics 13, 1689–1700. 10.2217/pgs.12.161 23171334

[B28] Lassmann H. (2018). Multiple sclerosis pathology. Cold Spring Harb. Perspect. Med. 8:a028936 10.1101/cshperspect.a028936 29358320 PMC5830904

[B29] Louapre C. Lubetzki C. (2015). Neurodegeneration in multiple sclerosis is a process separate from inflammation: Yes. Mult. Scler. 21, 1626–1628. 10.1177/1352458515587598 26515289

[B30] Mahurkar S. Moldovan M. Suppiah V. Sorosina M. Clarelli F. Liberatore G.. (2017). Response to interferon-beta treatment in multiple sclerosis patients: a genome-wide association study. Pharmacogenomics J. 17, 312–318. 10.1038/tpj.2016.20 27001119

[B31] Martínez A. de las Heras V. Mas Fontao A. Bartolomé M. de la Concha E. G. Urcelay E.. (2006). An IFNG polymorphism is associated with interferon-beta response in Spanish MS patients. J. Neuroimmunol. 173, 196–199. 10.1016/j.jneuroim.2005.12.002 16430971

[B32] Mitrovič M. Patsopoulos N. A. Beecham A. H. Dankowski T. Goris A. Dubois B.. (2018). Low-frequency and rare-coding variation contributes to multiple sclerosis risk. Cell 175, 1679–1687.e7. 10.1016/j.cell.2018.09.049 30343897 PMC6269166

[B33] Naito S. Namerow N. Mickey M. R. Terasaki P. I. (1972). Multiple sclerosis: association with HL-A3. Tissue Antigens 2, 1–4. 5077731 10.1111/j.1399-0039.1972.tb00111.x

[B34] Oksenberg J. R. Barcellos L. F. Cree B. A. C. Baranzini S. E. Bugawan T. L. Khan O.. (2004). Mapping multiple sclerosis susceptibility to the HLA-DR locus in African Americans. Am. J. Hum. Genet. 74, 160–167. 10.1086/380997 14669136 PMC1181903

[B35] Patsopoulos N. A. Baranzini S. E. Santaniello A. Shoostari P. Cotsapas C. Wong G. (2019). Multiple sclerosis genomic map implicates peripheral immune cells and microglia in susceptibility. Science 365:eaav7188 10.1126/science.aav7188 31604244 PMC7241648

[B36] Patsopoulos N. A. Barcellos L. F. Hintzen R. Q. Schaefer C. van Duijn C. M. Noble J. A.. (2013). Fine-mapping the genetic association of the major histocompatibility complex in multiple sclerosis: HLA and non-HLA effects. PLoS Genet. 9:e1003926. 10.1371/journal.pgen.1003926 24278027 PMC3836799

[B37] Patsopoulos N. A. Esposito F. Reischl J. Lehr S. Bauer D. Heubach J.. (2011). Genome-wide meta-analysis identifies novel multiple sclerosis susceptibility loci. Ann. Neurol. 70, 897–912. 10.1002/ana.22609 22190364 PMC3247076

[B38] Richard-Miceli C. Criswell L. A. (2012). Emerging patterns of genetic overlap across autoimmune disorders. Genome Med. 4:6. 10.1186/gm305 22284131 PMC3334554

[B39] Ross C. J. Towfic F. Shankar J. Laifenfeld D. Thoma M. Davis M. (2017). A pharmacogenetic signature of high response to copaxone in late-phase clinical-trial cohorts of multiple sclerosis. Genome Med. 9:50 10.1186/s13073-017-0436-y 28569182 PMC5450152

[B40] Sanna S. Pitzalis M. Zoledziewska M. Zara I. Sidore C. Murru R. (2010). Variants within the immunoregulatory CBLB gene are associated with multiple sclerosis. Nat. Genet. 42, 495–497. 10.1038/ng.584 20453840 PMC3786343

[B41] Sawcer S. Hellenthal G. Pirinen M. Spencer C. C. A. Patsopoulos N. A. Moutsianas L. (2011). Genetic risk and a primary role for cell-mediated immune mechanisms in multiple sclerosis. Nature 476, 214–219. 10.1038/nature10251 21833088 PMC3182531

[B42] The International Multiple Sclerosis Genetics Consortium, Hafler, D. A. Compston A. Sawcer S. Lander E. S. Daly M. J. (2007). Risk alleles for multiple sclerosis identified by a genomewide study. N. Engl. J. Med. 357, 851–862. 10.1056/NEJMoa073493 17660530

[B43] Tsareva E. Y. Kulakova O. G. Boyko A. N. Shchur S. G. Lvovs D. Favorov A. V. (2012). Allelic combinations of immune-response genes associated with glatiramer acetate treatment response in Russian multiple sclerosis patients. Pharmacogenomics 13, 43–53. 10.2217/pgs.11.136 22111603

[B44] Vidmar L. Maver A. Drulović J. Sepčić J. Novaković I. Ristič S. (2019). Multiple sclerosis patients carry an increased burden of exceedingly rare genetic variants in the inflammasome regulatory genes. Sci. Rep. 9:9171 10.1038/s41598-019-45598-x 31235738 PMC6591387

[B45] Vilariño-Güell C. Zimprich A. Martinelli-Boneschi F. Herculano B. Wang Z. Matesanz F. (2019). Exome sequencing in multiple sclerosis families identifies 12 candidate genes and nominates biological pathways for the genesis of disease. PLoS Genet. 15:e1008180 10.1371/journal.pgen.1008180 31170158 PMC6553700

[B46] Weber F. Fontaine B. Cournu-Rebeix I. Kroner A. Knop M. Lutz S.. (2008). IL2RA and IL7RA genes confer susceptibility for multiple sclerosis in two independent European populations. Genes Immun. 9, 259–263. 10.1038/gene.2008.14 18354419

